# Does zafirlukast reduce future risk of asthma exacerbations in adults? Systematic review and meta-analysis

**DOI:** 10.1186/2049-6958-9-30

**Published:** 2014-05-28

**Authors:** Chao Feng Chen, Yan Lv, Hong Ping Zhang, Gang Wang

**Affiliations:** 1 Pneumology Group, Department of Integrated Traditional Chinese and Western Medicine, West China Hospital, Sichuan University, Chengdu 610041, People’s Republic of China

**Keywords:** Asthma exacerbations, First-line and add-on therapy, Meta-analysis, Systematic review, Zafirlukast

## Abstract

**Background and objective:**

The purpose of asthma management is to achieve a total asthma control that involves current control and future risk. It has proven efficacy in reducing asthma exacerbations, but the effect size of zafirlukast for asthma exacerbations of various severity is not systematically explored.

**Methods:**

Randomized controlled trials were searched in PubMed Central, Web of Science, and Embase, where zafirlukast prevented asthma exacerbations in adults. The primary outcome was asthma exacerbations, the secondary outcomes were asthma exacerbations requiring systemic corticosteroids and emergency visits, respectively. Odds ratio (OR) with 95% confidence intervals (CI) were pooled.

**Results:**

Twelve trials were identified. As first-line therapy, compared to those having placebo, the patients with chronic asthma receiving zafirlukast experienced statistically lower asthma exacerbations (OR = 0.68, 95% CI = [0.45, 1.00]), but it was not found that zafirlukast was superior to placebo in asthma exacerbations requiring systemic corticosteroids (OR = 0.76, 95% CI = [0.45, 1.29]). Furthermore, zafirlukast was inferior to ICs in asthma exacerbations (OR = 2.11, 95% CI = [1.35, 3.30]) and requiring systemic corticosteroids (OR = 3.71, 95% CI = [1.82, 7.59]). As add-on therapy, zafirlukast was not superior to placebo in asthma exacerbations (OR =0.99, 95% CI = [0.54, 1.81] and requiring emergency visits (OR = 0.72, 95% CI = [0.18, 2.99]). Intriguingly, there was not a significant difference in asthma exacerbations between zafirlukast and ICs (OR = 1.12, 95% CI = [0.53, 2.34]).

**Conclusions:**

Our study suggests that zafirlukast, as the first-line therapy, significantly reduces mild to moderate but not severe asthma exacerbations. In the add-on regimen, zafirlukast could not reduce asthma exacerbations, which would perhaps result from small sample size and need**s** to be further studied.

## Introduction

Asthma is a chronic inflammatory disorder of airways closely associated with airway hyperresponsiveness. It is estimated that around 300 million people suffer from asthma and the burden of this disease to governments, health care systems, families, and patients is increasing worldwide [1,2]. Despite advances in knowledge of the pathophysiology of asthma and availability of effective therapy, currently asthma cannot be cured yet.

The goal of asthma treatment is to achieve and maintain asthma optimal control, which includes current control and long-term components referred to as “risk” or “future risk” since 2009 [3]. Asthma control is evaluated by a global assessment of asthma symptoms, reliever medicine use, lung function, and the frequency/severity of exacerbations. It is disappointing that only 2% of asthma patients are considered controlled across eight Asia-Pacific countries and Hong Kong in real world settings when control is assessed using the Global Initiative for Asthma (GINA) classification [4]. Asthma exacerbations, as the most important future risk, are recognized as a common clinical manifestation in patients with severe asthma, and are known to increase the risk of asthma mortality [5]. Hence, in recent years to prevent asthma exacerbations has been identified as an important component of establishing ideal asthma control in all asthma treatment guidelines. Inhaled corticosteroids (ICs) and combination of ICs/long-acting β_2_-agonists (LABA) are the mainstay of therapy for reducing asthma exacerbations including the severe ones, but some patients require additional treatment or prefer not to use ICs. In such patients, leukotriene receptor antagonist (LTRA), is a promising alternative to ICs therapy.

Although LTRA has been proven to be effective in improving asthma control in clinical practice, few studies systematically focused on effects of LTRA on future asthma outcomes, such as exacerbations and their various types. Furthermore, the relative effect size of LTRAs for prevention of future risk of asthma exacerbations remains unclear. Also because there is a great heterogeneity in pooled data of different types of LTRA, such as montelukast, zafirlukast, panlukastandzileuton [6]. This systematic review and meta-analysis is aimed to explore effects of zafirlukast, as first-line and add-on therapy, on prevention of asthma exacerbations in adults.

## Methods

### Search strategy and selection criteria

We searched electronic databases including PubMed (1991 to June 2013), Embase (1992 to June 2013) and Web of Science (1992 to June 2013) for randomized controlled trials (RCTs) using a comprehensive search strategy including the following keywords: “zafirlukast AND (asthma or wheezing), Limits Activated: Randomized Controlled Trial (RCT)”. We reviewed reference lists of all included studies, systematic reviews and narrative reviews to identify potentially relevant citations. There was no limitation on language or year of publication.

Trials were included if they met the following criteria: they were randomized controlled trials comparing zafirlukast versus placebo or other active drugs and reporting at least one asthma exacerbation. The trials were excluded if the patients were hospitalized for acute asthma because the primary outcome of this study for zafirlukast in treating asthma included hospitalization for asthma exacerbation. After exclusion of duplicates, two reviewers (C.F.C. and Y.L.) reviewed the full text of all citations with titles and abstracts that seemed to fit the criteria of inclusion. Irrelevant citations or not randomized controlled trials were not reviewed in full text. The number of citations rejected and the reasons for rejection were tracked.

### Data extraction and quality assessment

We reviewed each eligible citation, and obtained the full text of all definite of possible randomized controlled trials. From each article we extracted details regarding authors, year of publication, sex, sample size, sample size calculation, standard treatments, interventions as the first-line or add-on therapies, outcomes, adverse events, and intention to treat analysis and so on.

The methodological quality of included trials was assessed independently by two reviewers (CFC and YL). If information was not reported adequately, authors or sponsors of each included trial were contacted to acquire the accuracy of the methodology and primary data. The quality of the methods of each trial was assessed with the Cochrane Collaboration’s tool for assessing risk of bias [7]. Our judgments of high, low, and unclear risk of bias were corroborated by citations from trial reports, correspondence, or summarized information from the relevant sections of the individual study reports. Any disagreement between reviewers was resolved by consensus or by the third reviewer (G.W.).

### Primary and secondary outcomes

In the meta-analysis assessing the prevention of zafirlukast from asthma exacerbations, the number of asthma exacerbations was a priori specified primary outcome so as the number of detailed different exacerbations such as requiring systemic corticosteroids, whereas emergency visits and hospitalizations were identified as secondary outcomes.

### Statistical analysis, and assessment and evaluation of the evidence quality

The included trials were divided into two types of analyses stratified by zafirlukast as the first-line or the add-on therapies. The mean daily dose of ICs was converted in “microgram (μg) of beclomethasone equivalent”. Asthma exacerbations as dichotomous variables were reported as odds ratio (OR).

Heterogeneity was assessed by means of the Cochran Q method and by the test of inconsistency (I^2^). A random effects model was used if the Q statistic (p < 0.1) or I^2^ (> 50%) was significant, or we used a fixed effects model. We carried out subgroup analysis to assess the source of heterogeneity and we assessed the presence of publication bias visually with a funnel plot. Differences in bias risk and number needed to treat (NNT) were calculated to assess clinical significance. All estimates were reported with 95% confidence intervals (CI) and all p were 2-tailed. The meta-analysis was performed with Stata 11.0 (Stata Corp LP, College Station, Texas), and the risk bias of the methodological quality was assessed using RevMan 5.1 (Cochrane Review Manager, The Cochrane Collaboration, Oxford, UK).

The quality of the evidence related to the estimation of benefits and disadvantages in asthma exacerbation in adult population requiring oral/parenteral corticosteroid or emergency visits followed the suggestions of the GRADE Working Group (http://www.gradeworkinggroup.org/index.htm) by adopting the use of GradePro software 3.6 (http://ims.cochrane.org/revman/gradepro).

## Results

### Trials included, study characteristics and quality of reporting

**Figure 1:**
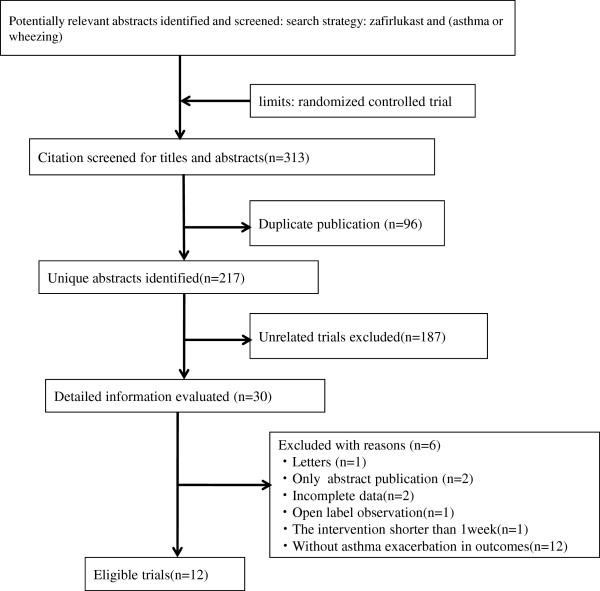
Flow diagram of trial selection and details of study identification, inclusion and exclusion.

The search strategy initially yielded 313 citations. Figure 1 shows details of study identification, inclusion and exclusion. Twelve unique trials with 4,398 participants which met the inclusion criteria came into statistical analysis [8-19]. All the trials recruited adults and adolescents. The characteristics of the included studies are listed in Table 1. The intervention period ranged from 4 to 48 weeks (median: 26 weeks). There were two types of interventions for zafirlukast, and they were first-line (n = 10) or add-on (n = 4) treatments. The dosage of zafirlukast was administrated 20 mg twice a day, except for two trials [14,19], where patients were treated with zafirlukast 80 mg twice daily, or were given 5 mg, 10 mg and 20 mg twice daily, respectively. The number of patients included in these studies varied between 38 and 762. In included studies with chronic persistent asthma, FEV_1_% of predicted value at baseline was more than 45%. In general, rescue β_2_-agonists were permitted.

**Table 1:** Characteristic of the included trials

**Study ID**	**Age mean ± SD [Range], y**	**Gender (F/M)**	**Intervention**	**Standard treatments**	**Compliance (%)**	**FEV**_**1**_**%**	**Run in period**	**Treatment duration**	**Type of intervention**	**Reported outcomes**		
										**AE**	**AES**	**AEE**
Boushey HA 2005 [8]	T: 33.6 ± 11.1	T: 47/29	T: Z 20 mg + IP bid	IB 800 ug bid × 10D or prednisone × 5D if asthma worsened	> 90	≥ 70	4 weeks	48 weeks	First-line/add-on	+	—	+
	C1: 33.2 ± 9.5	C1: 48/25	C1: IB 200 ug + OP bid									
	C2: 32.0 ± 10.5	C2: 43/33	C2: IP + OP bid									
Huang CJ 2003 [9]	T: 58.6 ± 3.0	T: 8/9	T: Z 20 mg bid	IB (≥ 400 μg/day or equivalent) + SABA	NR	Moderate	2 weeks	4 weeks	Add-on	+	—	+
	C: 56.9 ± 2.8	C: 7/7	C: Placebo 20 mg bid									
Brabson JH 2002 [10]	T: 35 ± 16	T: 141/75	T: Z 20 mg bid	Albuterol as needed	≥ 88	60-85	8 days	6 weeks	First-line	+	+	+
	C: 36 ± 14	C: 134/90	C: IFP 88 ug bid									
Nathan RA 2001 [11]	T: 32	T: 65/85	T: Z 20 mg bid	Albuterol as needed	NR	50-80	7-14 days	4 weeks	First-line	+	—	—
	C: 31	C: 79/65	C: Fluticasone 88 ug bid									
Busse W 2001 [12]	T: 12-75	T: NR	T: Z 20 mg + IP bid	Albuterol as needed, or oral or parental corticosteriods for AE	NR	50-80	8-14 days	12 weeks	First-line/add-on	+	+	—
	C: 12-75	C: NR	C1: IFP 88 ug + OP bid									
			C2: IP + OP bid									
Kim KT 2000 [13]	T: 32.9	T: 127/89	T: Z 20 mg + IP bid	Albuterol as needed	88	60- 85	1 week	6 weeks	First-line	+	+	+
	C: 35.5	C: 135/86	C: IFP 88 ug bid									
Virchow JCJr 2000 [14]	T: 47.4 ± 12.6	T: 85/95	T: Z 80 mg bid	Beclomethasone ≥ 1200 ug/day or equivalent) + SABA	T: 95	50-75	2 weeks	6 weeks	Add-on	+	—	—
	C: 49.2 ± 12.9	C: 98/90	C: Placebo 80 mg bid		C: 94							
Bleecker ER 2000 [15]	T: 31	T: 113/107	T: Z 20 mg bid	Albuterol as needed	92	50-80	8-14 days	12 weeks	First-line	+	—	—
	C: 31	C: 112/119	C: IFP 88 ug bid									
Busse W 1999 [16]	T: 36.9	T: 85/60	T: Z 20 mg bid	Albuterol as needed	NR	50-80	7-14 days	4 week	First-line	+	—	—
	C: 38.6	C: 77/67	C: SX 42 ug bid									
Nathan RA 1998 [17]	T: 33.2	T: 127/104	T: Z 20 mg bid	SABA as needed	NR	45-80	2-3 weeks	13 weeks	First-line	+	+	—
	C: 32.1	C: 132/91	C: Placebo 20 mg bid									
Fish JE 1997 [18]	T: > 12	T: 220/294	T: Z 20 mg bid	Albuterol as needed	NR	> 55	7-14 days	13 weeks	First-line	+	—	—
	C: > 12	C: 102/146	C: Placebo 20 mg bid									
Spector SL 1994 [19]	T: Z1: 37, Z2: 35, Z3: 36, C: 36	T: Z1: 18/52, Z2: 23/45, Z3: 18/50, C: 20/50	T: Z1: 20 mg bid, Z2: 10 mg bid, Z3: 5 mg bid, C: Placebo bid	Albuterol as needed	NR	40-75	2 weeks	6 weeks	First-line	+	—	—

AE, asthma exacerbations; AEE, asthma exacerbation require emergency visits; AES, asthma exacerbation require systematic corticosteroid; bid, twice daily; C, control group; D, days; F, female; IB, inhalation budesonide; IFP, inhalied fluticasone propionate; IP, inhalation of placebo; M, male; NR, not reported; OP, oral placebo; SABA, short β_2_ agonist; SD, standard deviation; SX, Salmeterolxinafoate; T, treatment group; y, year; Z, zafirlukast; +, positive report; —, negative report.

All the included trials were randomized and controlled. Table 2 shows an overview of the risk bias of each trial. There was no conflict of interest among eligible studies. Double blinding was used in all trials. On the whole, most of the included studies were of high methodological quality.

**Table 2:** Methodological quality of included studies

**Study**	**Randomly assigned**	**Allocation concealment**	**Multi- center**	**Blinding**	**Complete outcome data adequately addressed**	**Free of selective outcomes reporting**	**Other bias**	**Intention to treat**	**Sample size calculation**	**Power**	**Conflict of interest**
Boushey 2005 [8]	Yes	Yes	Yes	Double-blind	Yes	Unclear	Unclear	No	Yes	0.90	No
Huang 2003 [9]	Yes	Yes	No	Double-blind	Yes	Unclear	Unclear	No	No	NA	No
Brabson 2002 [10]	Yes	Yes	Yes	Double-blind	Yes	Unclear	Unclear	Yes	Yes	0.80	No
Nathan 2001 [11]	Yes	Yes	Yes	Double-blind	Yes	Unclear	Unclear	No	Yes	0.80	No
Busse 2001 [12]	Yes	Yes	No	Double-blind	Yes	Unclear	Unclear	No	Yes	0.80	No
Kim 2000 [13]	Yes	Yes	No	Double-blind	Yes	Unclear	No	Yes	Yes	0.80	No
Virchow 2000 [14]	Yes	Yes	Yes	Double-blind	Yes	Unclear	Unclear	No	No	NA	No
Bleecker 2000 [15]	Yes	Yes	Yes	Double-blind	Yes	Unclear	No	Yes	Yes	0.80	No
Busse 1999 [16]	Yes	Yes	Yes	Double-blind	Yes	Unclear	Unclear	Yes	Yes	0.80	No
Nathan 1998 [17]	Yes	Yes	Yes	Double-blind	Yes	Unclear	Unclear	No	Yes	0.90	No
Fish 1997 [18]	Yes	Yes	Yes	Double-blind	Yes	Unclear	Unclear	No	Yes	0.90	No
Spector 1994 [19]	Yes	Yes	Yes	Double-blind	Yes	Unclear	No	No	Yes	0.90	No

NA, not available.

### Outcomes for meta-analysis

#### First-line therapy

**Figure 2:**
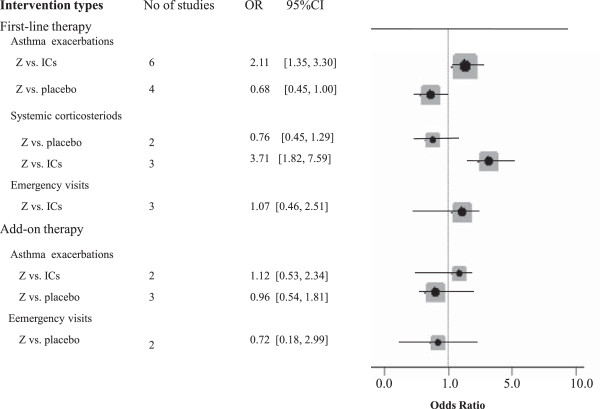
**Pooled odds ratio of patients experiencing different types of asthma exacerbations, comparing controlled drugs with zafirlukast.** Trials stratified according to controlled treatments (ICs and placebo) and intervention types. OR, odds ratio.

The primary and secondary outcomes are shown in Figure 2. Compared to those having placebo, the patients with chronic asthma receiving zafirlukast experienced statistically lower asthma exacerbations (OR = 0.68, 95% CI = [0.45, 1.00]). However, zafirlukast as the first-line therapy in chronic asthma was not superior to placebo in requiring systematic corticosteroids for asthma exacerbation (OR = 0.76, 95% CI = [0.45, 1.29]). Furthermore, zafirlukast was inferior to ICs in patients experiencing asthma exacerbations (OR = 2.11, 95% CI = [1.35, 3.30]) and asthma exacerbations requiring systematic corticosteroids (OR = 3.71, 95% CI = [1.82, 7.59]) but not in those requiring emergency visits (OR = 1.07, 95% CI = [0.46, 2.51]).

In placebo-controlled studies, only two trials which had met the inclusion criteria about patients requiring systematic corticosteroids for asthma exacerbations came into meta-analysis, so we could not perform subgroup and sensitivity analysis. Our subgroup and sensitivity analysis in ICs-controlled studies indicated that long-term ICs with more than 12 weeks did not get much more benefit in emergency visits in comparison to that with less than 12 weeks (OR = 2.56, 95% CI = [0.80, 8.20] vs. OR = 1.07, 95% CI = [0.46, 2.51]).

#### Add-on therapy

When zafirlukast was taken as an add-on therapy, we unexpectedly found it was not superior to placebo in asthma exacerbations (OR =0.99, 95% CI = [0.54, 1.81] and emergency visits (OR = 0.72, 95% CI = [0.18, 2.99]). Intriguingly, there was not a significant difference in asthma exacerbations between zafirlukast and ICs (OR = 1.12, 95% CI = [0.53, 2.34].

### Assessment and recommendation of zafirlukast for prevention of asthma exacerbations

The details are shown in Table 3. We assessed the asthma exacerbations and the participants who required emergency visits or needed systemic corticosteroids because of asthma exacerbations. According to the methodology of each trial, the quality of evidence of zafirlukast as first-line treatment compared with placebo, first-line/add-on treatment compared with ICs was of low or moderate quality. The trials failed to undertake intention-to-treat analysis and publication of bias in the funnel plot among the trials contributed to decrease the quality of evidence.

**Table 3:** Assessment of benefits and disadvantages in asthma exacerbation

**Clinical outcomes**	**Comparisons**	**Therapy type**	**Illustrative comparative risk**		**Relative effect (95% CI)**	**No. of participants (studies)**	**Quality of evidence (GRADE)**
			**With comparator**	**With intervention**			
Asthma exacerbation	Zafirlukast vs. placebo	First-line	92/1000	62/1000 (44 to 92)	OR = 0.68 [0.45, 1.00]	1,255 (n = 4)	⊕ ⊕ ⊝⊝ Low^1^
	Zafirlukast vs. ICs	First-line	31/1000	64/1000 (42 to 96)	OR = 2.11 [1.35, 3.30]	1,963 (n = 6)	⊕ ⊕ ⊕⊝ Moderate^2^
	Zafirlukast vs. ICs	Add-on	83/1000	92/1000 (46 to 175)	OR = 1.12 [0.53, 2.34]	353 (n = 2)	⊕ ⊕ ⊝⊝ Low^3^
	Zafirlukast vs. placebo	Add-on	121/1000	120/1000 (69 to 200)	OR = 0.96 [0.54, 1.81]	383 (n = 3)	⊕ ⊕ ⊝⊝ Low^4^
Asthma exacerbation requiring systemic corticosteriod	Zafirlukast vs. placebo	First-line	108/1000	86/1000 (40 to 186)	OR = 0.76 [0.45, 1.29]	544 (n = 2)	⊕ ⊕ ⊝⊝ Low^5^
	Zafirlukast vs. ICs	First-line	18/1000	64/1000 (32 to 123)	OR = 3.71 [1.82, 7.59]	1,089 (n = 3)	⊕ ⊕ ⊕⊝ Moderate^6^
Asthma exacerbation requiring emergency treatment	Zafirlukast vs. ICs	First-line	22/1000	23/1000 (10 to 53)	OR = 1.07 [0.46, 2.51]	994 (n = 3)	⊕ ⊕ ⊝⊝ Low^7^
	Zafirlukast vs. placebo	Add-on	60/1000	44/1000 (11 to 159)	OR = 0.72 [0.18, 2.99]	163 (n = 3)	⊕ ⊕ ⊝⊝ Low^8^

CI,= confidence intervals; ICs,= inhaled corticosteroids; OR, odds ratio.

1 (−1 limitations) (−1 publication bias). Four trials (Boushey HA 2005, Busse W 2001, Fish JE 1997, Nathan RA 1998) have high quality, but all of them fail to adhere to an intention-to-treat analysis, so suggesting high likelihood of bias (−1 of quality). Four trials were included, from the funnel plot we strongly suspected there was publication bias.

2 (−1 limitations). Busse W 2001 and Nathan RA 1998 fail to adhere to an intention-to-treat analysis, so suggesting high likelihood of bias (−1 of quality).

3 (−1 limitations) (−1 publication bias). Busse W 2001 and Boushey HA 2005 fail to adhere to an intention-to-treat analysis, so suggesting high likelihood of bias (−1 of quality). Only two trials were included, from the funnel plot we strongly suspected there was publication bias.

4 (−1 limitations) (−1 publication bias). Three trials (Boushey HA 2005, Busse W 2001, Huang CJ 2003) have high quality, but all of them fail to adhere to an intention-to-treat analysis, so suggesting high likelihood of bias (−1 of quality). Three trials were included, from the funnel plot we strongly suspected there was publication bias.

5 (−1 limitations) (−1 publication bias).Busse W 2001 and Nathan RA 1998 fail to adhere to an intention-to-treat analysis, so suggesting high likelihood of bias (−1 of quality). Only two trials were included, from the funnel plot we strongly suspected there was publication bias.

6 (−1 limitations) (−1 publication bias) (+1 large effect). Busse W 2001 fails to adhere to an intention-to-treat analysis, so suggesting high likelihood of bias (−1 of quality). Three trials were included, from the funnel plot we strongly suspected there was publication bias. Large effect (M-H pooled OR = 3.712) in the absence of other methodological limitations), so upgrading quality of evidence.

7 (−1 limitations) (−1 publication bias).Boushey HA 2005 fails to adhere to an intention-to-treat analysis, so suggesting high likelihood of bias (−1 of quality). Trials were included, from the funnel plot we strongly suspected there was publication bias.

8 (−1 limitations) (−1 publication bias).Boushey HA 2005 and Huang CJ 2003 fail to adhere to an intention-to-treat analysis, so suggesting high likelihood of bias (−1 of quality). Trials were included from the funnel plot we strongly suspected there was publication bias.

## Discussion

According to GINA report, the goal of asthma management is to achieve and maintain optimal asthma control [2]. Recently, the concept of asthma control has been extended to include an assessment of future risk in addition to the previous focus on the current impairment from asthma [20,21]. Despite advances in knowledge of the pathophysiology of asthma and via availability of effective therapy, asthma cannot currently be cured yet. As the most important factors in future risk of asthma, asthma exacerbations are common in asthmatic patients’ life, and constitute a major burden on health care resources. In patients with chronic mild to moderate asthma, zafirlukast significantly reduced asthma exacerbations in the first-line regimen when compared to placebo, but we did not find that zafirlukast was superior to placebo in asthma exacerbations in add-on regimen. Furthermore, zafirlukast was inferior to ICs in reducing asthma exacerbations and asthma exacerbations requiring systemic corticosteroids. It suggested that there was no absolute advantage from zafirlukast in relieving severe asthma attack such as asthma exacerbations requiring systemic corticosteroids or emergency visits.

Preventing recurrent exacerbations is an important goal in asthma therapy. Zafirlukast showed an overall beneficial effect on the symptoms of chronic asthma as measured by asthma exacerbations and other parameters when compared to placebo. Though there is a less-stringent definition for asthma exacerbation (based on patient’s current therapy change, oral or inhaled steroids use, worsening symptoms, emergency room treatment or hospitalization, or rescue albuterol use), definitions of a mild or moderate asthma exacerbation are justifiable according to a recently published ATS/ERS statement [20]. On the basis of ATS/ERS, severe asthma exacerbations can be defined as events that require urgent action on the part of the patient and physician to prevent a serious outcome, such as use of systemic corticosteroids, hospitalization or death from asthma; moderate exacerbations as events that result in a temporary change in treatment, in an effort to prevent the exacerbation from becoming severe; and mild exacerbations that are only just outside the normal range of variation in symptoms or changes in flow rates for the individual patient and may reflect transient loss of asthma control. In our study, the available data demonstrated that zafirlukast only decreased the risk of mild and moderate asthma exacerbations, but not severe asthma exacerbations, such as requiring systemic corticosteroids and emergency visits in chronic persistent asthma. Although zafirlukast was superior to placebo in reducing the risk of asthma exacerbations, our results indicated that patients receiving low-dose ICs from 352 to 400 ug daily had fairly fewer asthma exacerbations than those taking zafirlukast. It was consistent with Ducharme FM’s results that, in adults with mild to moderate chronic asthma, the risk of exacerbations requiring systemic glucocorticoids was 60% higher with daily oral leukotriene receptor antagonists than with doses of ICs equivalent to 400 ug/day inhaled beclometasone [6].

ICs were considered as the first-line treatment for patients with moderate to severe persistent asthma, but some patients require additional treatments or prefer not to use ICs. Our study manifested that the addition of zafirlukast to ICs could not reduce the asthma exacerbations as future risk of asthma outcomes. It indicated that zafirlukast, as an add-on therapy, might be possibly associated with improving asthma symptoms but not asthma exacerbations in future. Reid et al. found that zafirlukast significantly improved FEV_1_ and PEF, and reduced morning waking with asthma and β_2_-agonist puffs [22]. In bronchial challenge studies, zafirlukast attenuated the lower airway symptoms and pulmonary response to cat challenge [23,24]. Asthma is a complex multifactorial disorder involving a variety of different mechanisms. Environmental exposure and genetic background play a major role in development of asthma and triggering asthma symptoms. Genetic studies have produced heterogeneous results with little replication. More than 100 genes had been reported in connection to asthma or asthma phenotypes since the first study linking a genetic locus to asthma in 1989 [25]. Our recent study systemically explored the network of asthma-related genes where three hundred and twenty-six genes were identified [26]. The poor asthma control is related to deteriorative airway inflammation, asthma-related comorbidities such as obesity, rhinitis, gastroesophageal reflux disease, obstructive sleep apnoea, and exacerbation risk which is an important component to investigate asthma control [27].

In our systematic review, 10 out of twelve trials definitely described subjects characterized by no smoking, but there was not enough information to identify the status of smoking in other two trials [10,11]. Active smoking is associated with an increased morbidity from asthma and impairs the response to ICs [28,29]. The children exposed to environmental tobacco smoking had impaired recovery after hospitalization for acute asthma [30]. Extensive studies reveal that diet, particularly breast-feeding, is related to the development of asthma. Atopy is an independent risk factor of occupational asthma [31]. Body mass index (BMI) is an important independent predictor of asthma development and poor asthma control and asthma outcome [31], but we did not perform the subgroup analysis because only one trial provided the information on BMI [8]. More available data are needed to reveal the effect of zafirlukast on asthma exacerbations in patients with different BMI. Compared to normal weight asthmatics, the obese children are more likely to have severe disease and airflow obstruction [32].

There are sorts of inflammatory cells and mediators related to asthma mechanisms. An important role in airways inflammation is played by cysteinylleukotrienes, which are powerful agents inducing bronchoconstriction, mucus hypersecretion and airways hyperresponsiveness, and act as chemoattractants for eosinophils in the airways [33]. In patients with mild to moderate persistent asthma, zafirlukast, one of leukotriene receptor antagonists, produced rapid improvement in pulmonary function and daytime asthma symptoms, meanwhile decreasing the need for rescue therapy with β_2_-agonist and improving asthma control when combined with ICs [34]. Unexpectedly, we did not find that zafirlukast was superior to placebo in asthma exacerbations and emergency visits in either the first-line and add-on therapies. Furthermore, we did not find difference in asthma exacerbations between zafirlukast and ICs in add-on regimens. However, because of small sample size, it does not seem completely believable that zafirlukast was not superior to placebo in asthma exacerbations requiring systemic corticosteroids, and there was no significant difference in emergency visits for asthma exacerbations between zafirlukast and ICs in the first-line regimen. Additionally, all results from comparisons between zafirlukast, ICs and placebo in the add-on regimens were also somewhat unreliable for a low power. This suggests that more randomized controlled trials or larger sample size are needed to identify these difference among zafirlukast, ICs and placebo.

Our study has some limitations. First, the small number of trials could have lowered the power and lead to relative wide confidence intervals also precluding meta-regression analysis although high quality randomized controlled trials were included. Second, as it is well known, smoking, obesity, allergen, adherence and age are factors modifying the response to antiasthma medications, but this information was not available to be further analyzed.

## Conclusions

This systematic review included 12 randomized controlled trials to assess the efficiency of zafirlukast in preventing asthma exacerbations as future risk in chronic persistent mild to moderate asthma. Our results showed that zafirlukast could decrease risk of mild and moderate but not severe exacerbations as the first-line regimen. Furthermore, it was evidently inferior to ICs in any kinds of exacerbations. Because it has not enough available information to support subgroup and sensitivity analysis, more trials are needed to confirm these conclusions that would benefit the asthma patients in clinical practice.

## Abbreviations

ATS: American Thoracic Society; CI: Confidence interval; ERS: European Respiratory Society; FEV_1_: Forced expiratory volume in one second; FEV_1_% of predicted: Predicted of forced expiratory volume in one second percentage; GRADE: Grading of Recommendations Assessment, Development, and Evaluation; GINA: Global Initiative for Asthma; ICs: Inhaled corticosteroids; LTRAs: Leukotriene receptor antagonists; OR: Odds ratio; RCTs: Randomized controlled trials.

## Competing interests

The authors declare that they have no competing interests.

## Authors’ contributions

CFC contributed to collect, analyze the data, and write the paper. YL and HPZ participated in the collection of data. GW participated in the design of the study and analysis, and reviewed the manuscript. All authors read and approved the final manuscript.
